# Structural and Biophysical Characterization of Stable Alpha-Synuclein Oligomers

**DOI:** 10.3390/ijms232314630

**Published:** 2022-11-23

**Authors:** Nishant Vaikath, Indulekha Sudhakaran, Ilham Abdi, Vijay Gupta, Nour Majbour, Simona Ghanem, Houari Abdesselem, Kostas Vekrellis, Omar El-Agnaf

**Affiliations:** 1 Neurological Disorder Research Center, Qatar Biomedical Research Institute (QBRI), Hamad Bin Khalifa University (HBKU), Qatar Foundation, Doha P.O. Box 5825, Qatar; 2 Center of Basic Research, Biomedical Research Foundation of the Academy of Athens, 11527 Athens, Greece

**Keywords:** alpha-synuclein, oligomers, HNE, ONE, DA, Parkinson’s disease

## Abstract

The aggregation of α-synuclein (α-syn) into neurotoxic oligomers and fibrils is an important pathogenic feature of synucleinopatheis, including Parkinson’s disease (PD). A further characteristic of PD is the oxidative stress that results in the formation of aldehydes by lipid peroxidation. It has been reported that the brains of deceased patients with PD contain high levels of protein oligomers that are cross-linked to these aldehydes. Increasing evidence also suggests that prefibrillar oligomeric species are more toxic than the mature amyloid fibrils. However, due to the heterogenous and metastable nature, characterization of the α-syn oligomeric species has been challenging. Here, we generated and characterized distinct α-syn oligomers in vitro in the presence of DA and lipid peroxidation products 4-hydroxy-2-nonenal (HNE) and 4-oxo-2-nonenal (ONE). HNE and ONE oligomer were stable towards the treatment with SDS, urea, and temperature. The secondary structure analysis revealed that only HNE and ONE oligomers contain β-sheet content. In the seeding assay, both DA and ONE oligomers significantly accelerated the aggregation. Furthermore, all oligomeric preparations were found to seed the aggregation of α-syn monomers in vitro and found to be cytotoxic when added to SH-SY5Y cells. Finally, both HNE and ONE α-syn oligomers can be used as a calibrator in an α-syn oligomers-specific ELISA.

## 1. Introduction

The accumulation of aggregated forms of α-synuclein (α-syn) in neuronal and non-neuronal cells in the brain is a hallmark of synucleinopathies including Parkinson’s disease (PD), dementia with Lewy bodies (DLB), and multiple system atrophy (MSA) [1]. In PD and DLB, α-syn accumulates in neuronal cells as Lewy bodies (LBs) and Lewy neurites (LNs), whereas in MSA, α-syn typically accumulates in glial cytoplasmic inclusions (GCIs) in the oligodendrocytes. Under physiological conditions, α-syn is a 140-amino acid presynaptic protein that exists in a disordered conformation or an α-helical, membrane-associated, multimeric conformation [2]. Under pathological conditions, α-syn converts into β-sheets that stack to form protofibrils and eventually to mature amyloid fibrils [2,3]. The deposition of aggregated forms of α-syn is a critical step in developing synucleinopathies [3,4,5,6]. However, the exact cause for α-syn to convert to its pathogenic aggregated form is still unknown and it is currently attributed to various factors including increased levels of α-syn due to gene multiplications and mutations, impaired mitochondrial function, and its association with membranes [2]. In addition to α-syn mutations, α-syn aggregation is also influenced by various posttranslational modifications including phosphorylation, ubiquitination, nitration, and oxidation which have been identified in experimental models as well as brains from PD patients [7,8,9].

There is indeed tangible evidence suggesting a strong relationship between insoluble protein aggregates and neurodegeneration. However, multiple findings support the notion that it is the soluble intermediate oligomeric species that are the most neurotoxic rather than the insoluble fibrillar aggregates [10,11,12,13,14,15]. Although there are significant advances made towards understanding the structure of fibrillar aggregates, comparatively less information is available regarding the structural characteristics of soluble oligomeric species. One of the biggest challenges being the process of isolating these oligomers in a stable form. This is in fact due to the nature of these oligomers, which are extremely labile and short-lived. Once these oligomers are formed and reach a threshold concentration; they are rapidly converted into mature fibrils [16]. Studies are currently focused on stabilizing the intermediate oligomeric species formed during the aggregation process using various small compounds, metals, lipids, and molecular chaperons. However, some additive-induced oligomer preparations were also shown to be non-toxic as they do not seed the aggregation process; a prerequisite for toxicity [17,18]. Moreover, it has also been a challenge to produce a sufficient amount of the toxic oligomeric intermediates to study their structural characteristic and toxicity.

Impaired antioxidant mechanisms in the ageing brain may lead to oxidative stress resulting from the generation of reactive oxygen species (ROS), and such pathological events have been found to promote the aggregation of α-syn in vitro [19,20]. One such event includes the redox reactions of the neurotransmitter dopamine (DA) in the nigral neurons, which lead to the generation of superoxide and hydrogen peroxide [20]. DA has been shown to promote stable oligomers both in vitro and in neurons [21,22,23,24]. Moreover, the interaction of α-syn with an oxidized form of DA is suggested to be responsible for its toxicity [25,26,27,28]. Due the selective vulnerability of the dopaminergic neurons in PD and related disorders, DA-mediated α-syn oligomerization is pathophysiologically relevant. At excess physiological levels, ROS present in the neuronal cell membrane can also initiate the lipid peroxidation of polyunsaturated fatty acids leading to the formation of reactive aldehydes including 4-oxo-2-nonenal (ONE) and 4-hydroxy-2-nonenal (HNE) [29,30]. HNE and ONE being cytotoxic by themselves are also found to covalently modify α-syn monomers into soluble oligomers [29,31,32]. Moreover, increased levels of protein adducts formed with these aldehydes have been demonstrated in PD with 58% of PD nigral neurons consisting of HNE-modified proteins compared to 9% controls [33].

In this study, we prepared distinct α-syn oligomers generated in vitro in the presence of DA, HNE, or ONE. We compared and characterized their structural, biophysical, and functional properties by a combination of thioflavin-T (Th-T) fluorescence, electron microscopy, circular dichroism, Congo red binding assay, in vitro seeding assay, toxicity in cells, and their usability as calibrators in ELISA.

## 2. Results

### 2.1. Preparation of Stable α-Syn Oligomers

**Figure 1:**
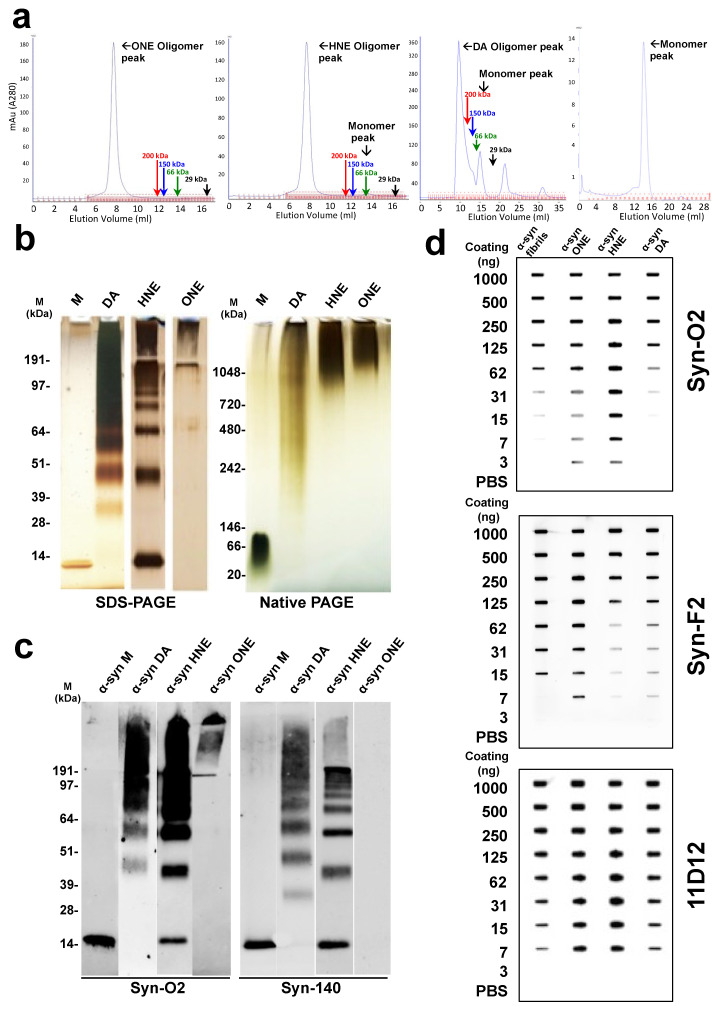
The purification and characterization of α-syn oligomers. (**a**) Purification of α-syn DA, HNE and ONE oligomers by SEC. α-Syn incubated in the presence of DA, HNE, or ONE was loaded onto a Superdex 200 (10/300) column and eluted at a flow rate of 0.5 mL/min in PBS and elution monitored at 280 nm. The elution profile of the standards Dextran blue (2000 kDa); β-amylase (200 kDa); alcohol dehydrogenase (150 kDa); albumin, bovine serum (66 kDa) and carbonic anhydrase (29 kDa) are indicated by an arrow. (**b**) Silver staining under denaturing SDS-PAGE (left) and non-denaturing Native PAGE (right). Two micrograms of α-syn monomers or oligomers were loaded onto the gel and silver-stained. (**c**) Western blot analysis of the oligomers under denaturing conditions. 50 ng of α-syn monomers or oligomers was loaded onto SDS-PAGE gel, transferred to a nitrocellulose membrane, and probed with mouse anti-α-syn monoclonal antibody, Syn-O2 or polyclonal sheep antibody, Syn-140. (**d**) Slot blot results showing the reactivity of conformational antibodies Syn-O2 or Syn-F2 and generic antibody 11D12 to different quantities of α-syn fibrils and α-syn ONE, HNE, or DA oligomers.

The preparation and purification of α-syn oligomers was challenging owing to their inherently transient and heterogeneous nature. Nevertheless, multiples procedures were carried out to overcome these hurdles and to generate stable oligomers. In the present study, we utilized the ability of certain additives to cross-link α-syn monomers to form stable oligomers. Here, we utilized the ability of HNE, ONE, and DA to cross-link to α-syn monomers to generate stable oligomers, characterized them using various biochemical and biophysical methods, and finally tested their toxicity in cells. The incubation of α-syn monomers with HNE or ONE for 18 h resulted in the formation of high -molecular-weight species as evident by the FPLC chromatogram (Figure 1a). This was also confirmed by silver staining using Native-PAGE gel where both HNE and ONE oligomer showed a smear of >1000 kDa (Figure 1b). Even though the addition of ONE resulted in the complete crosslinking of the monomers to high-molecular-weight oligomers, incubation with HNE did show a small fraction of α-syn monomers (Figure 1a). The FPLC chromatogram for DA oligomers showed a broader peak of >200 kDa (Figure 1a) along with a peak corresponding to a monomeric fraction Figure (Figure 1a). Using Native-PAGE silver staining, the high-molecular-weight DA oligomers were found to behave as a smear with a size ranging from >200 kDa to >1000 kDa, indicating the presence of both low- and high-molecular-weight oligomers (Figure 1b). A-Syn monomers eluted with a single peak correspond to molecular weight of ~60 kDa (Figure 1a). This is in accordance with the previous reports where they found that monomeric α-syn behaves like a large protein of ~50–60 kDa when assessed by non-denaturing techniques including size exclusion chromatography and native-PAGE gels [34].

### 2.2. ONE-, HNE- and DA- Oligomers Display Size Differences and Greatly Differ in Their Oligomer Conformation

In order to determine the nature of these oligomers we carried out silver staining using SDS-PAGE and Native PAGE gels. In SDS-PAGE gel, the HNE-induced oligomers appear as multiple low- and high-molecular-weight bands including a monomeric band (Figure 1b), whereas the ONE-induced oligomers appear only as a high-molecular-weight smear confined to the stacking gel but no monomeric band (Figure 1b). On the contrary, the DA-induced oligomers showed a high-molecular-weight smear and a few-low-molecular weight bands without any monomeric band (Figure 1b). The analysis of the samples of the silver staining SDS-PAGE gel indicates that the HNE-induced oligomers are not stable under SDS and heating conditions, as they tend to break up into monomers and other high-molecular-weight oligomers whereas the ONE-induced oligomers are stable under these conditions, giving a single smearing band at the stacking region of the gel. Then, we analyzed the size distribution of these samples by running them in native gel. Interestingly, both the HNE- and ONE-induced oligomers appeared as smear with a molecular weight corresponding to >1000 kDa (Figure 1b). In contrast, the DA-induced oligomers appeared as smear with an apparent molecular weight of >200 kDa (Figure 1b).

Then, we detected these oligomers by Western blot using conformation-specific antibody Syn-O2 and an in-house sheep polyclonal antibody Syn-140. Consistent with the silver staining data, Syn-O2 detected high- and low-molecular weight bands from HNE-, ONE, and DA oligomers (Figure 1c). On the other hand, Syn-140 recognized less of the high-molecular-weight bands from DA- and HNE-oligomers (Figure 1c). Surprisingly, Syn-140 did not recognize any band from the ONE-oligomers. It is possible that the Syn-140 antibody failed to recognize ONE oligomers because the epitopes were masked by their conformation (Figure 1c).

In order to determine the conformational specificity of the oligomers, we utilized a slot blot assay using different antibodies. The oligomers were coated onto a nitrocellulose membrane to sub-nanogram levels and detected using conformation-specific antibodies (Syn-O2, Syn-F2) and a pan antibody (11D12). Syn-O2 was able to detect both ONE- and HNE-oligomers up to 3 ng, but showed lower affinity for the DA-oligomers. The Syn-F2 antibody showed higher affinity to only ONE-oligomers than HNE-and DA-oligomers. We also confirmed the proper loading of each α-syn oligomer via in-house pan antibody 11D12 (Figure 1d).

### 2.3. ONE-, HNE-, and DA-Oligomers Display Size Differences and Greatly Differ in Their Oligomer Conformation

**Figure 2:**
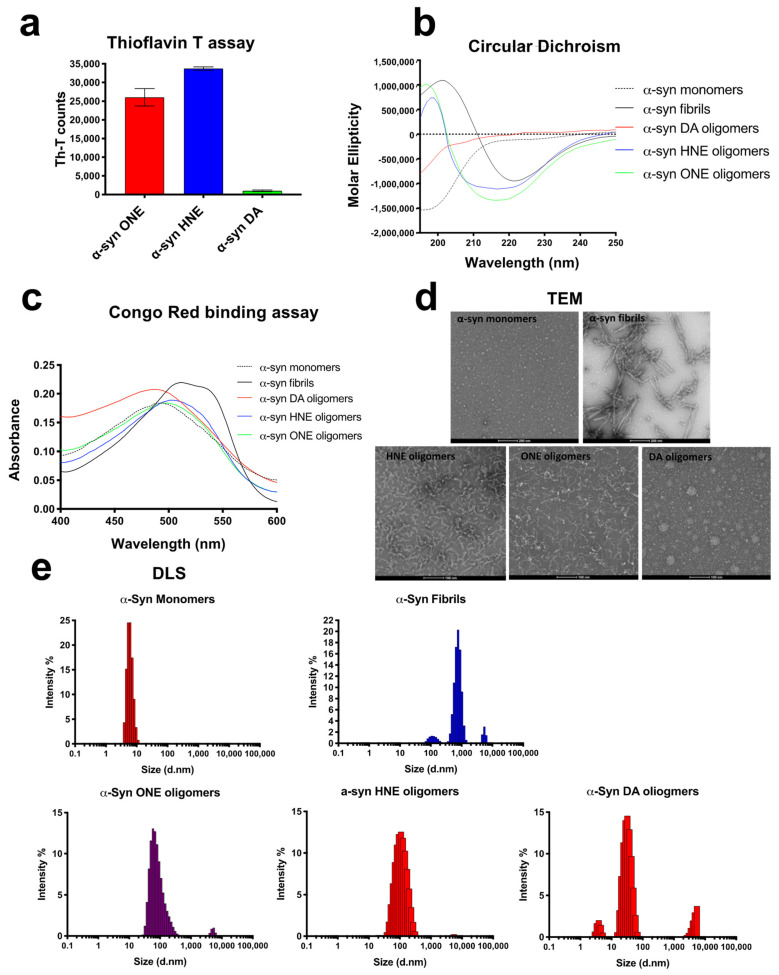
Biophysical characterization of α-syn oligomers. (**a**) Th-T assay: 5 μM α-syn oligomers were mixed with 20 μM Th-T. (**b**) Circular dichroism using 5 μM of α-syn monomers, fibrils, and different oligomers (**c**) Congo red binding assay: 5 μM α-syn monomers, fibrils, or oligomers were mixed and incubated with 20 μM of Congo red dye for 30 min and absorbance measured between 400 and 600 nm. (**d**) Negatively stained TEM images of α-syn monomers, fibrils (100 nm), and oligomers (scale bars, 200 nm). (**e**) Dynamic light scattering using 5 μM α-syn monomers, fibrils, and oligomers expressed as intensity with hydrodynamic diameter on the *x* axis.

We then investigated whether the three oligomer preparations differed in their secondary structure. We first checked whether they showed any signal for the amyloid dye Th-T, a dye commonly used for detecting β-sheet-rich conformers. Both HNE- and ONE-oligomers showed high Th-T fluorescence, whereas the DA-oligomers showed negligible Th-T fluorescence (Figure 2a). This was also confirmed by circular dichroism (CD) spectroscopy, and Congo red binding assay. The CD spectra showed that both HNE- and ONE- oligomers possessed extensive β-sheet conformation as apparent by the negative minimum absorption near 218 nm and a positive maximum near 200 nm (Figure 2b). α-Syn fibrils was included as a positive control for the extensive β-sheet conformations and α-syn monomers as a negative control with no beta-sheet conformation. DA-oligomers did not show any β-sheet conformations as with α-syn monomers. Finally, the presence of β-sheet was further confirmed by Congo red (CR) binding assay. A shift in absorbance maxima of the CR red dye from 490 nm to 508 nm indicates the presence of increased cross β-sheet rich structure in α-syn [32]. Consistent with the CD assay, both HNE- and ONE- oligomers showed a shift in the CR absorption spectra compared to the CR alone (Figure 2c). α-Syn DA-oligomers, on the other hand, did not show any shift indicating the absence of any β-sheet conformation. Furthermore, the morphology of the oligomers was assessed by TEM (Figure 2d). DA oligomers were found to contain both small and large spherical structure, whereas the HNE and ONE oligomers were found to comprise small spherical bead-like structures resembling a string (Figure 2d). Furthermore, using dynamic light scattering, the most abundant particles present in ONE, HNE, and DA appeared to have a Z-average size of 141 ± 4.5 nm, 98 ± 2.35 nm, and 22 ± 16 nm (from the cumulate analysis of three repeated measurements of the same sample) and a polydispersity index of 0.25 ± 0.03, 0.19 ± 0.01, and 0.56 ± 0.25, respectively (Figure 2e).

### 2.4. Stability

**Figure 3:**
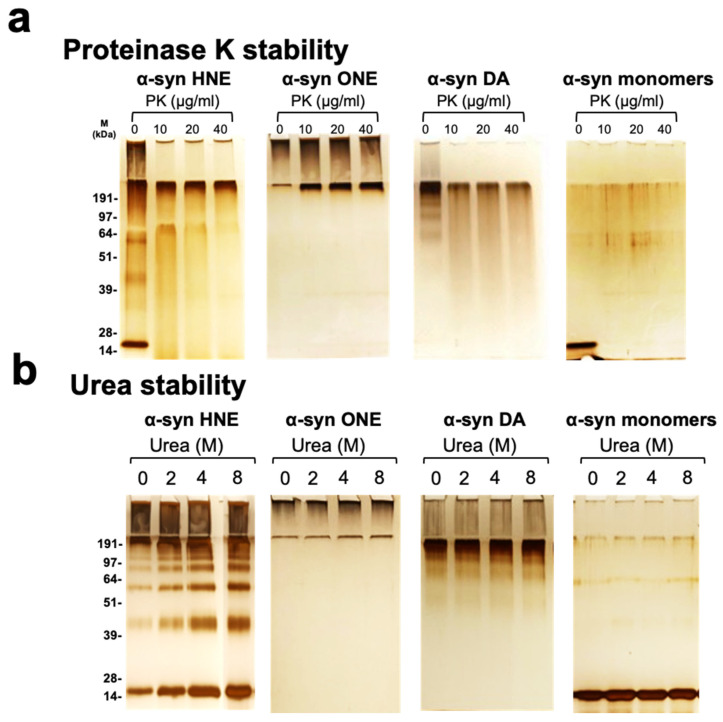
Stability of oligomers. (**a**) α-Syn monomers and different oligomers preparations were incubated alone (0 ug/mL) or with increasing concentrations of proteinase K (10, 20, or 40 μg/mL) at 37 °C for 30 min and loaded onto the gel and silver-stained. (**b**) α-Syn monomers and different oligomer preparations were incubated alone (0 M) or with 2, 4, or 8 M urea for 1 h at room temperature and loaded onto gel and silver-stained.

In order to study the stability of the oligomers, we performed multiple experiments including treating the samples with urea or proteinase K, the influence of temperature and the effect of freeze–thaw cycles. Treating the oligomeric samples with an increasing concentration of proteinase K showed differences in the stability of the oligomer preparations by silver staining. A-Syn HNE-induced α-synuclein oligomers were found to be resistant to proteinase K at a lower concentration, as evident by a smearing band that is comparable to the untreated sample. However, upon higher concentration of proteinase K, no bands were observed (Figure 3a). α-Syn ONE-induced oligomers were found to be resistant at a lower as well as higher concentration of proteinase K (Figure 3a). On the other hand, α-syn DA-induced oligomers were highly sensitive to proteinase K treatment and did not show any band, even at a lower concentration of proteinase K (Figure 3a). Furthermore, the oligomer preparations were treated with increasing concentrations of urea and its effect was checked by silver staining. All three oligomer preparations were found to be of varying stability with an increasing concentration of urea (Figure 3b). The HNE oligomers were found to be more sensitive to urea, as indicated by an increase in intensity of the low- and high-molecular-weight bands (Figure 3b). In contrast, the ONE oligomers were confined to the stacking gel and with the increase in urea concentration, the intensity of high-molecular-weight smear also increased (Figure 3b). This is in accordance with the previous report where they have shown that with increase in urea concentration, the intensity of high molecular weight band also increases [32]. In DA oligomers, the variation in intensity of bands with increase in urea were less pronounced.

**Figure 4:**
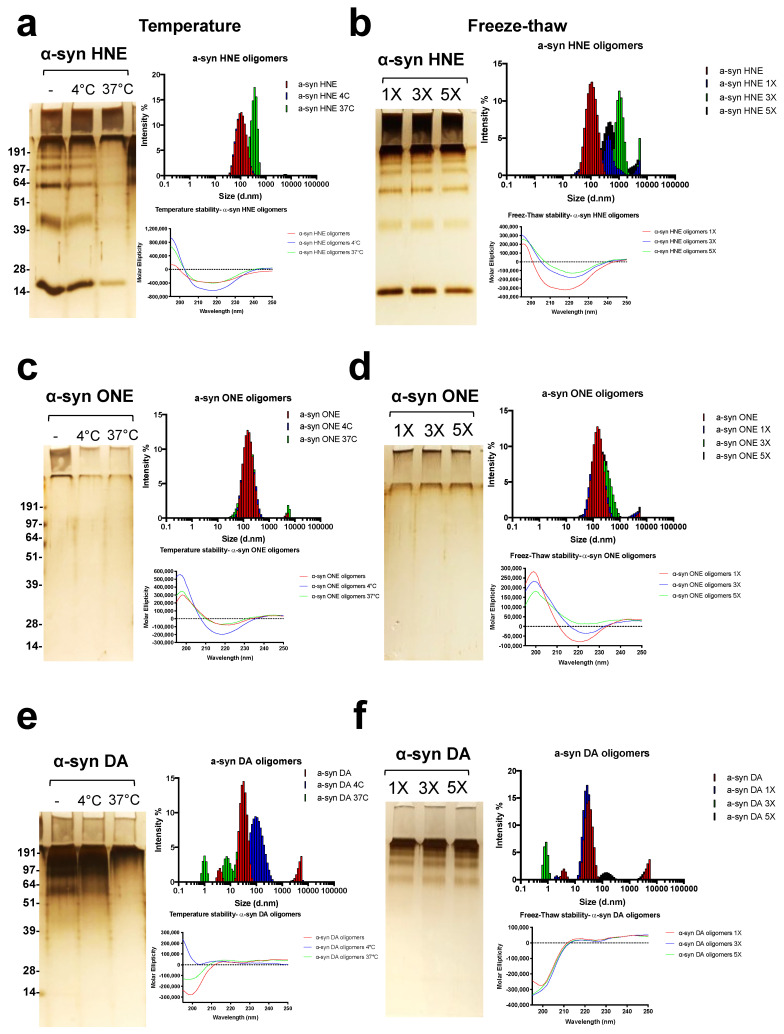
Effect of temperature and freeze–thaw cycles on the stability of oligomers: 5 μM of either α-syn HNE, -ONE, or -DA oligomers were incubated at 4 °C or 37 °C for 5 days (**a**,**c**,**e**) or subjected to multiple freeze–thaw cycles (**b**,**d**,**f**) and analyzed by silver-staining circular dichroism and dynamic light scattering experiment.

The stability of the oligomers was finally checked by incubation at different temperature and multiple freeze–thaw cycles. α-Syn HNE oligomers were found to be stable when incubated for 5 days at 4 °C but not at 37 °C as evident from the silver staining and DLS data (Figure 4a). When incubated at 37 °C, the DLS data clearly indicate the formation of additional higher-molecular-weight species with an average size of ~300 nm and the CD showing an increased positive maximum at ~198 nm (Figure 4a). Moreover, α-syn HNE oligomers had a drastic effect on exposure to multiple freeze–thaw cycles. This was made clearly visible by DLS measurement which showed the presence of additional high-molecular-weight species with an average size of more than ~300 nm, which was not observed by silver staining or the CD measurement (Figure 4b). It was interesting to note that α-syn ONE oligomers were found to be stable when treated at different temperatures as well as when exposed to multiple freeze–thaw cycles. The DLS measurement clearly showed that the α-syn ONE oligomers stayed as high-molecular-weight species when incubated at different temperature or at multiple freeze–thaw cycles (Figure 4c,d). On the other hand, the α-syn DA oligomers were found to be sensitive to both temperature and freeze–thaw cycles, as evident from the silver staining, and the CD and DLS measurements showing a heterogeneous nature (Figure 4e,f). In conclusion, α-syn ONE oligomers were found to be more stable compared to HNE or DA oligomers.

### 2.5. ONE and DA-Oligomers Can Seed the Aggregation of α-Syn Monomers at a Late Stage in an In Vitro Fibrillation Assay

**Figure 5:**
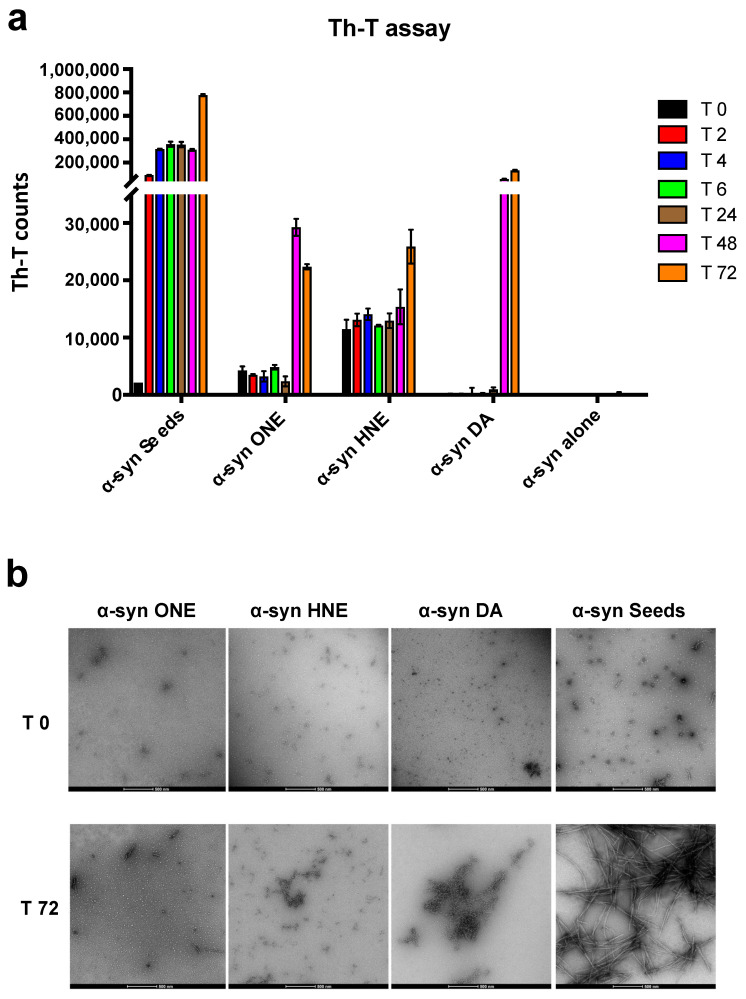
In vitro seeding assay. (**a**) Th-T assay for samples collected at different time points (as indicated) for α-syn monomers (25 μM) incubated alone or with 5 uM α-syn seeds or different oligomers at 37 °C, 800 rpm. (**b**) TEM images for samples collected at different time points (T 0 and T 72). Scale bar—200 nm.

Next, we wanted to see whether the oligomers could seed the aggregation of α-syn. It has been well-established that small aggregates or seeds of α-syn can accelerate the aggregation by a process known as “seeding” both in vitro and in vivo [35,36]. Moreover, these seeding assays have been widely used to study the inhibition of aggregation by certain compounds [37,38]. Here, we incubated the oligomer preparations with α-syn monomers and monitored the aggregation process by Th-T assay. We found that incubation of both DA- and ONE-oligomers significantly accelerated the aggregation of α-syn after 48 h of incubation as indicated by an increased Th-T signal, whereas the HNE-oligomers only had an effect at 72 h (Figure 5a). Even though there was an acceleration of aggregation by the oligomers, the effect was much lower compared to the α-syn seeds (Figure 5a) where the effect could be observed within 2 h. The acceleration of aggregation of α-syn by the oligomers in the later stage of the fibrillation assay indicates that they seed the already formed oligomers rather than the initial monomers. The morphology of the formed aggregate was also visualized and confirmed by TEM (Figure 5b)

### 2.6. Toxicity of α-Syn Oligomers

**Figure 6:**
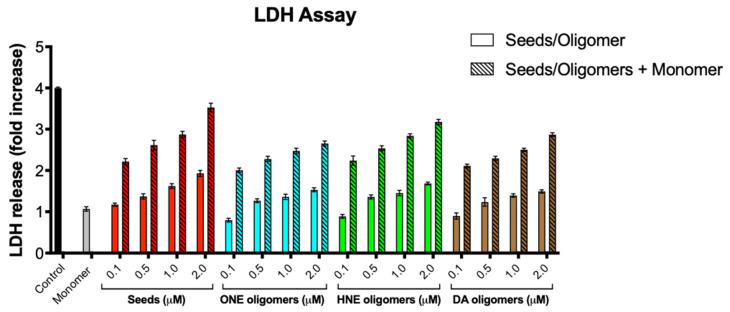
Toxicity of oligomers. LDH assay for cell apoptosis in SHY-5Y cells shows the presence of different concentrations (as indicated) of α-syn DA, HNE, and ONE oligomers incubated alone (solid bars) or in combination with 10 μM of α-syn monomers (dashed bars) after 48 h incubation.

Next, we assessed whether the stable oligomers generated have toxic effect on SH-SY5Y neuroblastoma cells. Our group previously established an assay-based α-syn mediated cellular toxicity assay wherein SH-SY5Y cells incubated with α-syn seeds followed by the addition of α-syn monomers led to aggregation resulting in cell toxicity [38]. Here, we incubated SH-SY5Y cells with various concentrations of oligomers and measured the toxicity by LDH assay. Upon incubation of SH-SY5Y cells with the oligomers alone, we found that only DA, ONE, and HNE oligomers were toxic at a concentration as low as 0.5 μM and higher (Figure 6). Moreover, when SH-5YSY cells were incubated with the oligomers along with α-syn monomers, the toxicity was significantly increased, suggesting that oligomers have the propensity to seed α-syn monomers (Figure 6).

### 2.7. α-Syn Oligomers as Calibrators in ELISA

**Figure 7:**
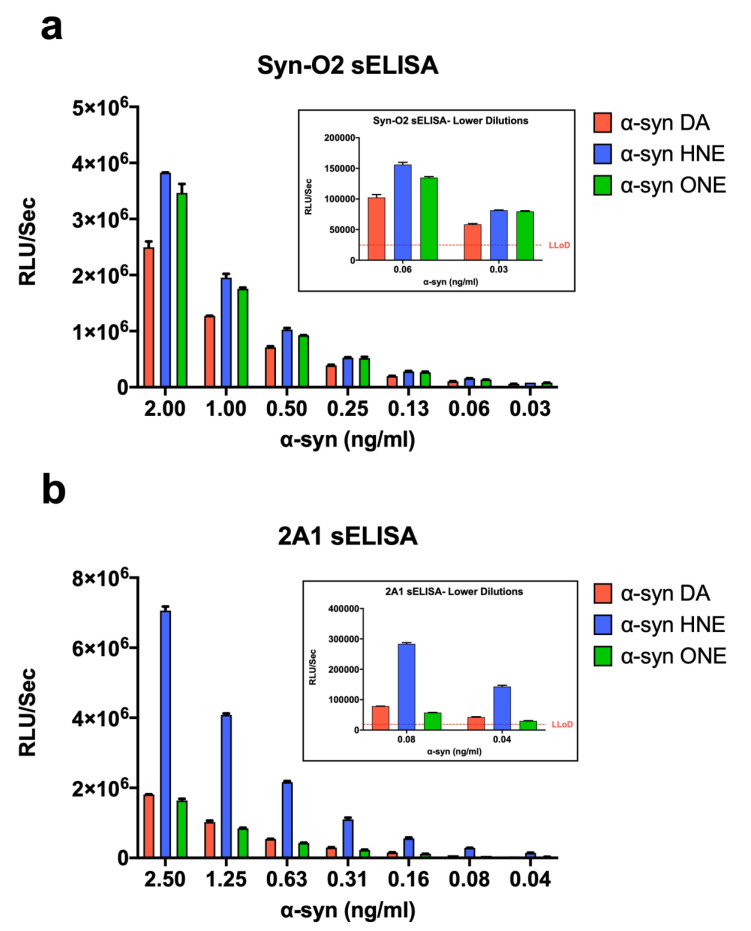
Oligomers as calibrators in a sandwich ELISA (sELISA). ELISA plates coated with (**a**) Syn-O2 (mouse anti-o-α-syn monoclonal antibody) or (**b**) 2A1 (mouse anti-α-syn aggregate-specific monoclonal antibody) was incubated with different concentration (as indicated) of α-syn DA-, HNE-, or ONE-oligomers followed by the addition of a biotinylated antibody and detected with streptavidin–peroxidase polymer. The chemiluminescence expressed in relative light units (RLU/s) was measured by adding an enhanced chemiluminescence substrate to the plate then measured immediately using the VICTOR™ X3 multilabel plate reader.

Next, we wanted to see whether these oligomers could be used as calibrators in ELISA. We compared the oligomers in our previously established ELISA [39] using α-syn antibody Syn-O2 or 2A1 as a capture antibody. The sensitivity of the assay using all three oligomers was found to be 30 pg/mL and 40 pg/mL using Syn-O2 and 2A1 antibody, respectively (Figure 7a,b). However, α-Syn HNE and ONE oligomers were found to be more sensitive at lower dilution compared to the α-syn DA oligomers, making HNE and ONE oligomers better calibrators than the DA oligomers. It is also interesting to see that the signal with HNE oligomers using the 2A1 antibody is much higher than the ONE and DA oligomers (Figure 7b). This might be the result of an epitope exposed in the HNE oligomers that are detected by 2A1 antibody.

## 3. Discussion

The aggregation of α-syn, leading to the intracellular Lewy body formation, has been considered as a critical process inducing toxicity and neurodegeneration in synucleinopathies. Accumulating evidence suggests that the toxicity of α-syn is not confined to its aggregated form but also with the soluble oligomeric forms that are released into the extracellular medium and transported across neurons [10,11,12,13,14,15]. Moreover, α-syn oligomeric species were detected and quantified in biological fluids including serum and cerebrospinal fluid and post-mortem brain tissues of patients with synucleinopathies [39,40,41,42,43]. Although these findings support that α-syn oligomeric species may play a key role in neurodegeneration, it is still unknown how they exert their toxicity. However, a recent study suggests that toxic oligomers have two structural elements that affect their ability to permeabilize biological membranes. The first element is an exposed lipophilic region that promotes the interaction with the membrane surface. The second is an oligomeric core rich in β-sheets that can insert into the membrane bilayer and disrupt it [44].

It has also been reported that due to elevated cellular oxidative stress, there is a considerable increase in lipid peroxidation in the brains of deceased PD patients [45]. Aldehydes such as HNE and ONE are generated under such conditions in vivo [46,47,48,49] and have been readily found to cross-link to proteins via Cys, His, and Lys residues and impair protein function [50]. Moreover, increased levels of HNE conjugated proteins were found in PD nigral neurons compared to the controls [33].

Recently, numerous in vitro approaches have been established to study the oligomeric state of α-syn. These in vitro preparations of α-syn oligomers were found to be mostly heterogeneous in nature with varying shapes and sizes that differed greatly based on the method of preparation [18,21,44,51,52,53,54,55,56,57,58,59,60,61,62]. It is generally assumed that α-syn leads to disease through a toxic gain-of-function by forming intermediate oligomers. However, there is no clarity in terms of what qualifies as a “toxic oligomer” unless defined based on its effect on the viability of cultured cells [63]. Consequently, many preparations of α-syn oligomers were reported as toxic or non-toxic [17,64,65,66,67,68]. However, due to the limited knowledge and understanding of the in vivo oligomers, it is still a challenge to identify which preparation would actually resemble the in vivo-formed oligomeric species.

Here, we utilized DA as well as the lipid peroxidation metabolite HNE and ONE to generate stable α-syn oligomers, compared their properties using biochemical and biophysical methods, and verified their use as calibrators in an immunoassay.

The incubation of α-syn monomers with a molar excess of HNE or ONE resulted in the complete conversion to oligomers yielding almost 100% soluble high-molecular-weight species, with the exception of α-syn HNE oligomers showing a very small percentage of monomers as observed from the FPLC data (Figure 1a). However, in SDS-PAGE, α-syn HNE and ONE oligomers showed a different migration profile. α-Syn HNE oligomers showed multiple bands ranging between 14 kDa and > 200 kDa, whereas α-syn ONE oligomers only showed a high-molecular-weight smear of >200 kDa. As previously reported, this might be attributed to the stability of the α-syn ONE oligomers in SDS compared to the α-syn HNE oligomers [32]. This was also confirmed when the samples were run under native conditions in the absence of SDS (Figure 1b). On the contrary, the α-syn DA oligomers gave rise to a heterogeneous mixture of species with a range of molecular weight species (Figure 1b), as observed in the FPLC data (Figure 1 a) and SDS-PAGE (Figure 1b) consistent with the previously reported data [18]. It is noteworthy to mention that in SDS-PAGE gel, the α-syn DA oligomers did not show any band corresponding to the monomers, as observed with the α-syn HNE oligomers. This in-turn indicates that the α-syn HNE oligomers are broken down into monomers as well as high-molecular-weight oligomers upon treatment with SDS. The heterogeneous nature of the α-syn DA oligomers was also confirmed by running the sample under native conditions. As shown in Figure 1b, the α-syn DA oligomers ran as a smear with a molecular weight >150 kDa. The size range observed for the different oligomer preparation agrees well with the size distribution of α-syn oligomers generated using similar approaches [69]. Moreover, electron microscopy images showed that the α-syn DA oligomers gave rise to species with a variety of sizes and shapes, which is in accordance with the previously reported study [21].

The structural property determination using CD gave an insight into the secondary structure of oligomers. α-Syn DA oligomers did not show any secondary structure similar to untreated α-syn monomers. It has been reported that incubation of α-syn with DA produces different kinds of species including α-syn oligomers, α-syn dimers and trimers in which the dimeric and trimeric species are found to have some β-sheet and turn content [58]. However, in our α-syn DA preparation, we did not find any beta-sheet content. The possible explanation could be the percentage of dimers and trimers in the sample compared to the high-molecular-weight oligomers. The α-syn HNE and ONE oligomers on the other hand showed high β-sheet content as evident by the Th-T (Figure 2a) and a negative minimum at ~218 nm in CD analysis (Figure 2b). This is well in accordance with the previously reported studies [32]. A potential contribution to synucleinopathy pathology might be attributed to α-syn DA-, HNE-, or ONE- oligomers, which may cause loss of neurons as well as the formation of aldehydes as a result of lipid peroxidation by reactive oxygen species [31,33,70].

Among the other characteristics, the ability to induce toxicity in the cells is an important feature of all amyloid oligomers [71]. In our study, we found that all the oligomer preparations were toxic to the cells when used at a concentration above 0.1 μM and the toxicity increased when the cells were seeded with oligomers along with α-syn monomers. Toxic α-syn aggregates are thought to propagate by seeding the conversion of native α-synuclein into pathological conformations and the addition of α-syn in vitro [37,72]. Several mechanisms have been proposed for α-syn oligomer-induced toxicity, including the disruption of membranes, endoplasmic reticulum stress, mitochondrial dysfunction, impairment of proteosomal, and the autophagic degradation and induction of neuroinflamation [63]. α-Syn DA oligomers have been shown to impair chaperone-mediated autophagy and SNARE complex formation in vitro [73,74]. Alternatively, the observed toxicity of the oligomers might be attributed to its ability to disrupt the lipid vesicles as α-syn oligomers were previously reported to bind brain-derived and synthetic phospholipid membranes and permeabilize synthetic vesicles [69,71,75,76]. The sites of interaction between dopamine and α-synuclein likely include residue E83 and the 125-YEMPS-129 motif in the C-terminus, since the mutation or deletion of these sites restores the ability of α-synuclein to form mature amyloid fibrils in the presence of dopamine [22,25,77]

α-Syn fibrils have been shown to play a central role in the progression of synucleinopathies. However, accumulating evidence suggests that the intermediate oligomeric species are certainly the deciding factor leading to the collapse of the neuronal homeostasis. In order to develop disease-modifying strategies targeting misfolded α-syn, it is imperative to detect and quantify the levels of toxic oligomeric species of α-syn present in biological fluids from patients with synucleiopathies at an early stage. Thus, characterizing stable α-syn oligomers is necessary not only to understand the structural basis of oligomers’ toxicity, but also to develop oligomer-specific diagnostic assays and therapeutic interventions in synucleinopathies.

In summary, a thorough knowledge on the formation, structure, and toxicity of α-syn oligomers is of the utmost importance for our understanding of α-syn misfolding in synucleinopathies, and may serve as a basis for developing specific and sensitive diagnostic assays as well as a means of therapeutic intervention. Despite having many differences in the morphology as well as stability, there are structural and functional similarities between the oligomer preparations that make them ideal candidates to be used in immunoassays. All three oligomer preparations studied here were found to be cytotoxic, hence, they could be of pathological relevance to PD and related disorders. The HNE-, ONE-, and DA-oligomers studied here could therefore be used as relevant calibrators in immunoassays that most likely mimic the oligomeric species accumulated during α-syn aggregation in patients with PD and other synucleinopathies.

## 4. Materials and Methods

### 4.1. Expression and Purification of α-Syn

Full-length recombinant α-syn was expressed and purified as previously reported [78]. Briefly, α-syn was expressed in *Escherichia coli* BL21 (DE3) using the bacterial expression vector pRK172. Following expression and sedimentation, the bacterial pellets from 1 L of TB broth were homogenized and sonicated in 50 mL of high-salt buffer (0.75 M NaCl, 10 mM Tris, pH 7.6, 1 mM EDTA) containing a cocktail of protease inhibitors (Thermo Scientific), heated to 100 °C for 10 min, and centrifuged at 5300× *g* for 20 min. The solution was dialyzed overnight against the buffer used for gel filtration chromatography (50 mM NaCl, 10 mM Tris, pH 7.6, 1 mM EDTA), following which the volume was reduced to 5 mL using a Pierce protein concentrator (10 K MWCO, Thermo Scientific, Rockford, IL, USA) according to the manufacturer’s instructions. All proteins were purified by size exclusion using a Superdex 200 gel filtration column (GE Healthcare, Chicago, IL, USA). The clean fractions were pooled, exchanged with a buffer (10 mM Tris pH 7.6, 25 mM NaCl, 1 mM EDTA, and 1 mM PMSF) for ion exchange chromatography by dialysis overnight, and were applied onto a HiTrap Q column (GE Healthcare, Chicago, IL, USA) and eluted in 10 mM Tris pH 7.6 using a linear gradient of 0.025–1.0 M NaCl. Purified fractions were pooled, and protein concentrations were determined using the Pierce BCA protein assay kit (Thermo Scientific, Rockford, IL, USA).

### 4.2. Generation of 4-Oxo-2-Nonenal (ONE)-, 4-Hydroxy-2-Nonenal (HNE)-, and Dopamine (DA)-Oligomers

HNE-/ONE- α-syn oligomers were prepared using previously reported protocol [32]. Briefly, α-syn was dialyzed against 50 mM disodium hydrogen phosphate, pH 8.5 followed by filtration using 100-kDa MWCO micron spin filter (Millipore) to remove high-molecular-weight aggregates. HNE or ONE was then added to α-syn monomers (140 μM) to obtain a final molar ratio of 30:1 (HNE/ONE: α-syn) followed by the incubation of the samples at 37 °C for 18 h without shaking. The samples were centrifuged at 16,900× *g* for 5 min and the oligomeric species generated were separated by size exclusion chromatography on a Superdex 200 gel filtration column (GE healthcare, Chicago, IL, USA) equilibrated with 20 mM Tris pH 7.4, 0.15 M NaCl buffer. The eluted peaks fractions corresponding to the oligomeric fraction was pooled and quantified using the BCA protein assay kit after solubilizing the oligomers in equal volume on 6 M GnHCl.

DA-α-syn oligomer was prepared according to a previously described protocol [18]. Briefly, α-syn was dialyzed against 50 mM Tris pH 7.5, 0.15 M KCl followed by filtration using 100-kDa MWCO micron spin filter. α-Syn (140 μM) was incubated with DA (Sigma) at a molar ratio of 1:10 (α-syn: DA) at 37 °C, 300 rpm for 3 days. The samples were centrifuged at 12,000× *g* for 5 min to remove any aggregates and α-syn oligomeric species generated were separated from the monomeric form of the protein by size exclusion chromatography on a Superdex 200 gel filtration column (GE healthcare, Chicago, IL, USA) equilibrated with PBS pH 7.4 buffer. The eluted peak fractions corresponding to the high molecular weight were pooled and taken for characterization. For measuring concentration, oligomers were solubilized in an equal volume of 6 M Gn-HCl and quantified using Pierce BCA protein assay kit (Thermo Scientific, Rockford, IL, USA)

### 4.3. Silver Staining

Two micrograms (2 μg) of the samples was loaded on to 15% SDS-PAGE gel and silver-stained using Biorad silver staining kit (BioRad, Hercules, CA, USA). To run the sample under non-denaturing conditions, 2 ug of the samples were loaded onto 4–16% Bis-Tris Native PAGE gel (ThermoFisher Scientific, Waltham, MA, USA) and run according to the manufacturer’s instructions and silver-stained.

### 4.4. Western Blotting

Fifty nanograms (50 ng) of the samples was loaded on to 15% SDS-PAGE gel. The proteins were subsequently transferred into a nitrocellulose membrane and Western transfer performed at 90 V for 1 h. The membrane was boiled in PBS for 5 min and blocked with 5% skimmed milk in PBST (0.05% Tween-20 in PBS) for 1 h at RT followed by incubation with primary antibodies. After washing with PBST, the membrane was incubated for 1 h with HRP-conjugated goat anti-mouse or HRP- conjugated goat anti-rabbit IgG (1/20,000 in PBST, Jackson ImunoResearch Laboratories Inc, Baltimore Pike, PA, USA). Following an additional washing step with PBST, the membrane was developed using a super signal West Pico chemiluminescent substrate (Pierce Biotechnology, Rockford, IL, USA).

### 4.5. Slot Blot Assay

The slot blot system was assembled with a pre-wet 0.2 μm nitrocellulose membrane following the manufacturer’s protocol (Fisher Scientific, Waltham, MA, USA). Specified amount of antigen prepared in 50 μL PBS was applied to each slot. Following this, the wells were washed with 1000 μL PBS and the membrane was air-dried for 45 min. The dried membrane was blocked with 5% skimmed milk in PBST for 1 h at RT. After blocking, the membranes were incubated in the respective primary antibodies (Syn O2, Syn F2 or 11D12 (50 ng/mL) overnight at 4 °C. Following washes with PBST, the membranes were incubated in a secondary antibody (Goat-anti mouse IgG-HRP; 1:20,000) for 1 h at RT. After final washes, the membranes were developed using West Pico chemiluminescent substrate and imaged using Biorad Imaging System.

### 4.6. In-Vitro Seeding Assay

α-Syn monomers (25 uM) were incubated with or without 5 μM seeds or different oligomers at 37 °C, 800 rpm for 48 h. Aliquots of samples were collected at time points 0, 2, 4, 6, 24, and 48 h and a Thioflavin-T assay was carried out.

### 4.7. LDH Activity Assay

SH-SY5Y cells were plated at a density of 15,000 cells/well in 96-well plate (Nunc), and incubated for 24 h at 37 °C, 5% CO_2_. Increasing concentrations (0.1, 0.5, 1, and 2 μM) of α-syn seeds, HNE-, ONE- or DA- oligomers in 100 µL of Opti-MEM were added to the cells in two sets. After 1 h of incubation, 10 µM α-syn monomers in 100 µL Opti-MEM were added to the cells in one set, and to the other set, 100 µL Opti-MEM was added and incubated for further 48 h. Control wells consisted of Opti-MEM with no treatment, and blank wells consisted of no cells. LDH activity was spectrophotometrically measured at 490–620 nm using a Pierce LDH Cytotoxicity Assay kit (Thermo Scientific, Rockfor, IL, USA) according to the manufacturer’s indications using 50 μL media from treated cells. LDH activity from untreated cells media was defined as a value of 1 and then, the amount of LDH released to the extracellular medium was expressed as fold increase of this control value in all the other samples. Statistical significance for the comparison was analyzed by one-way analysis of variance (ANOVA) with Bonferroni correction applied to attenuate the false positives arising from multiple comparison using Graphpad Prism software.

### 4.8. TEM

Samples for electron microscopy images were prepared by depositing the samples on Formvar-coated 400-mesh copper grids followed by fixing briefly with 0.5% glutaraldehyde and negatively stained with 2% uranyl acetate. Images were acquired using FEI Talos 200C electron microscope.

### 4.9. Circular Dichroism (CD)

One micromolar (1 μM) of α-syn monomers, fibrils, seeds and different oligomers was used for CD measurement. The far-UV CD spectra (between 250 and 190 nm) were recorded on a Chirascan™ CD Spectrometer (Applied Photophysics, Surrey, UK) with a step size of 0.1 nm using a cuvette with of path length of 1 mm. Each scan was repeated 5 times and the background value of the buffer was subtracted for all samples.

### 4.10. Congo Red

Five micromolar (5 μM) of samples was mixed with 20 μM of Congo red and incubated at RT for 30 min and an absorbance scan was carried out from 400 to 600 nm.

### 4.11. Dynamic Light Scattering (DLS)

DLS measurements were performed on a Zetasizer NANO ZS (Malvern Instruments Ltd., Worcestershire, UK) instrument. The samples were scanned using capillary cell (Malvern Instruments Ltd., Worcestershire, UK) at 5 μM. Every sample was measured three times and the averaged intensity-size distribution is reported.

### 4.12. Stability Test

Urea and Proteinase K stability studies

Five micrograms (5 μg) of α-syn monomers or different oligomer preparations was incubated alone (0 M) or with increasing concentrations of urea (2, 4, or 8 M) and incubated for 1 h at room temperature. Similarly, for the proteinase K treatment, 5 μg of α-syn monomers or different oligomer preparations was incubated alone (0 μg/mL) or with increasing concentrations of proteinase K (10, 20, or 40 μg/mL) at 37 °C for 30 min. Two micrograms (2 μg) of the urea or proteinase K treated samples was run on a 12% gel and silver-stained.

### 4.13. Oligomers as Calibrators in ELISA

Oligomeric α-syn specific sandwich-based ELISA [39] was used to compare the DA-, HNE-, ONE- crosslinked α-syn oligomers. Briefly, a 384-well black microplate (Nunc MaxiSorb, NUNC) was coated (50 µL/well) overnight at 4 °C with 0.2 µg/mL Syn-O2 (mouse anti-o-α-syn monoclonal antibody) or 2A1 antibody in 200 mM of sodium bicarbonate buffer, pH 9.6. Plate was then washed with PBST (PBS containing 0.05% Tween-20), blocked with blocking buffer (2.5% gelatin and 5% BSA in PBST) at 100 µL/well for 2 h at 37 °C and then washed with PBST. The α-syn oligomers were prepared as standards by serially diluting them in artificial CSF and then added to the plate (50 µL/well) and incubated for 2 h at 37 °C with shaking (40 rpm). After washing the plate with PBST, biotinylated pan-α-syn monoclonal antibody was added to the plate (50 µL/well) and incubated for 2 h at 37 °C. The plate was then washed again with PBST and streptavidin–peroxidase polymer, ultrasensitive (Sigma) diluted 1:5000 in blocking buffer was then added to the plate (50 µL/well) and incubated for 1 h at 37 °C. After washing, the chemiluminescence expressed in relative light units was measured by adding (50 µL/well) enhanced chemiluminescence substrate (SuperSignal ELISA Femto, Pierce Biotechnology, Rockford, IL, USA) to the plate, then immediately measured using VICTOR™ X3 multilabel plate reader (PerkinElmer, Waltham, MA, USA).

## Data Availability

Not applicable.
